# Gut resistome of NSCLC patients treated with immunotherapy

**DOI:** 10.3389/fgene.2024.1378900

**Published:** 2024-08-07

**Authors:** Ewelina Iwan, Anna Grenda, Arkadiusz Bomba, Katarzyna Bielińska, Dariusz Wasyl, Robert Kieszko, Anna Rolska-Kopińska, Izabela Chmielewska, Paweł Krawczyk, Kamila Rybczyńska-Tkaczyk, Małgorzata Olejnik, Janusz Milanowski

**Affiliations:** ^1^ Department of Omics Analyses, National Veterinary Research Institute, Pulawy, Poland; ^2^ Department of Pneumology, Oncology and Allergology, Medical University in Lublin, Lublin, Poland; ^3^ Department of Environmental Microbiology, University of Life Science, Lublin, Poland; ^4^ Department of Basic and Preclinical Sciences, Faculty of Biological and Veterinary Sciences, Nicolaus Copernicus University in Torun, Torun, Poland

**Keywords:** resistome, microbiome, NSCLC, immunotherapy, metagenomics

## Abstract

**Background:**

The newest method of treatment for patients with NSCLC (non-small cell lung cancer) is immunotherapy directed at the immune checkpoints PD-1 (Programmed Cell Death 1) and PD-L1 (Programmed Cell Death Ligand 1). PD-L1 is the only validated predictor factor for immunotherapy efficacy, but it is imperfect. Some patients do not benefit from immunotherapy and may develop primary or secondary resistance. This study aimed to assess the intestinal resistome composition of non-small cell lung cancer (NSCLC) patients treated with immune checkpoint inhibitors in the context of clinical features and potentially new prediction factors for assessing immunotherapy efficacy.

**Methods:**

The study included 30 advanced NSCLC patients, 19 (57%) men and 11 (33%) women treated with first- or second-line immunotherapy (nivolumab, pembrolizumab or atezolizumab). We evaluated the patient’s gut resistome composition using the high sensitivity of targeted metagenomics.

**Results:**

Studies have shown that resistome richness is associated with clinical and demographic factors of NSCLC patients treated with immunotherapy. Smoking seems to be associated with an increased abundance of macrolides, lincosamides, streptogramins and vancomycin core resistome. The resistome of patients with progression disease appears to be more abundant and diverse, with significantly higher levels of genomic markers of resistance to lincosamides (*lnuC*). The resistance genes *lnuC*, *msrD*, *ermG*, *ap*h(6), *fosA* were correlated with progression-free survival or/and overall survival, thus may be considered as factors potentially impacting the disease.

**Conclusion:**

The results indicate that the intestinal resistome of NSCLC patients with immune checkpoint inhibitors treatment differs depending on the response to immunotherapy, with several distinguished markers. Since it might impact treatment efficacy, it must be examined more deeply.

## Introduction

The newest method of treatment for patients with NSCLC (non-small cell lung cancer) is immunotherapy directed at the immune checkpoints PD-1 (Programmed Cell Death 1) and PD-L1 (Programmed Cell Death Ligand 1). Patient eligibility for treatment with immune checkpoint inhibitors (ICIs) is based on PD-L1 expression measured immunohistochemically on tumor cells. PD-L1 is the only validated predictor factor of immunotherapy efficacy, but it is not ideal. PD-L1/PD-1-based therapy (pembrolizumab, atezolizumab, cemiplimab, nivolumab) is effective and prolongs patients’ life. However, primary or secondary resilience against immunotherapy is observed in some patients (Topalian et al., 2019; West et al., 2019). Some patients with a high percentage of tumor cells with PD-L1 expression develop primary resistance to immunotherapy and progress (Gandhi et al., 2018). Another patient achieves disease stabilization with a short progression-free survival (PFS) (Gandhi et al., 2018). These patients develop an acquired resilience to immunotherapy (Gandhi et al., 2018; Topalian et al., 2019; West et al., 2019).

The background of the immunotherapy treatment failures is not fully understood. So far, except for PD-L1 expression on tumor cells, no predictive factors that will predict the effectiveness of immunotherapy have been identified (West et al., 2019). Resilience to immunotherapy is related to the molecular background, the activity of the immune system, the tumor microenvironment, genetic and epigenetic changes, and the composition of the intestinal microbiome (Boyero et al., 2020; Kovács and Győrffy, 2022; Liu et al., 2022; Vu et al., 2022). However, the impact of the whole microbiome (bacteria taxonomic composition, their interaction, genomic setup, including resistance genes - resistome) on NSCLC treatment is still a poorly understood phenomenon (Lurienne et al., 2020; Sun et al., 2023; Li et al., 2024). In particular, using antibiotics to control and prevent infections - related or irrelevant to NSCLC- may deplete the intestinal microbiota (Lurienne et al., 2020). Recent research shows that metagenomics studies, in aspects of taxonomic composition bacteria population and antibiotic resistance genes, may expose the existence of an association between the gut microbiome of NSCLC patients and their response to treatment (Grenda et al., 2022; Sun et al., 2023; Xi et al., 2023).

Antimicrobial resistance (AMR) hampers treatment efficacy and remains a significant health concern in human and veterinary medicine (Ferri et al., 2017; Irfan et al., 2022). National and international organizations such as World Health Organization (WHO) and European Centre for Disease Prevention and Control (ECDC) recognize AMR as a global threat and recommend prudent antimicrobial usage and systematic surveillance of AMR (https://www.ecdc.europa.eu/en/publications-data/surveillance-antimicrobial-resistance-europe-2022). Infection with resistant/multi-resistant pathogens and or prevalence of resistance in commensal bacteria that might become a reservoir of antibiotic resistance genes (ARGs) may have serious health consequences, especially in the case of immunocompromised patients (Irfan et al., 2022; Bonnet et al., 2023). Furthermore, the impact of resistance genes persisting in the microbiome on the progress and success of oncological therapy remains largely unknown (Sun et al., 2023; Li et al., 2024).

Contemporary AMR monitoring methods are mostly culture-based. The approach is biased due to a narrow perspective of a random cultured strain thai is not necessarily representative of the whole microbiome. Implementing high-throughput sequencing and metagenomics has become a game changer in monitoring and detecting genomic bases of resistance mechanisms and their mobilization potential (Likotrafiti et al., 2018). Direct metagenomics, while not without limitations, enables scientists to characterise resistance from the perspective of a single strain and the whole bacteria population - resistome (Flint et al., 2023). The main problems with direct metagenomics are high costs and low sensitivity for gene detection - only the most abundant genes may be detected (Lanza et al., 2018).

Therefore, we propose using custom, targeted metagenomics for a more comprehensive assessment of the resistome composition, mainly low-abundance acquired AGRs. Our study aimed to assess the composition of gut resistome of NSCLC patients treated with immune checkpoint inhibitors in the context of clinical features and potentially new predictive factors to evaluate the effectiveness of immunotherapy.

## Materials and methods

**FIGURE 1:**
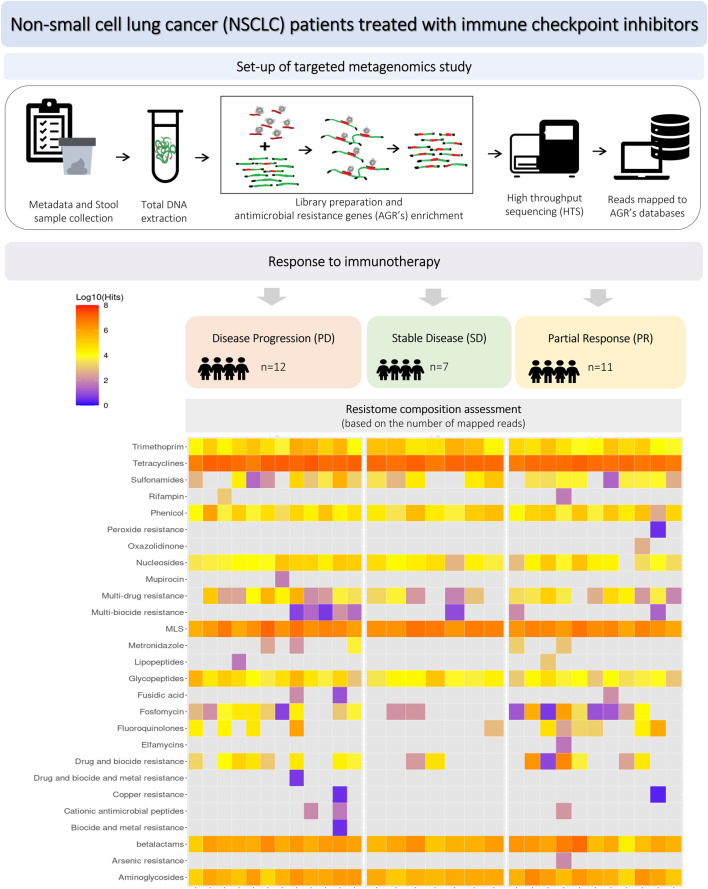
Workflow of the experiment, including study groups, design of metagenomics analysis and resistome profiling.

The workflow of the study is presented in Figure 1, including the design of experimental groups, the schema of the applied metagenomics approach and resistome profiling.

### Patients characteristic

The study included 30 NSCLC patients, 19 (57%) men and 11 (33%) women treated with first- or second-line immunotherapy (nivolumab, pembrolizumab or atezolizumab) from January 2019 to December 2020. The median age was 65.5 years (min-max: 48–76, SD = 7.1). Twenty-four patients (80%) were diagnosed with stage IV and six with stage IIIB NSCLC. Twenty patients (67%) were diagnosed with adenocarcinoma (AC), and 10 (33%) with squamous cell carcinoma (SqC). Seven patients (23%) were treated with pembrolizumab in first-line and 23 (67%) with nivolumab or atezolizumab in second-line. PD-L1 expression was assessed by immunohistochemistry (IHC) in the routine diagnostic procedure. PD-L1 was examined using an SP 263 antibody clone on Benchmark GX autostainer (Ventana Medical Systems, Inc, United States). Seven patients (23%) were treated with antibiotics prior to immunotherapy. Eleven patients (37%) were antibiotics treated during immunotherapy, of which six people were from the group treated up to 4 weeks before the initiation of immunotherapy. Eight patients (27%) were treated with proton pump inhibitors. Detailed characteristics of the study group are included in Table 1a/b.

**TABLE 1:** Clinical and demographic characteristics of patients qualified for the study for dichotomous (a) and continuous variables.

a) Dichotomous variables			
	Antibiotic usage up to 4 weeks before immunotherapy n (%)		
Factor	No n = 23 (77%)	Yes n = 7 (23%)	
Age			
Below the median (n = 15, 50%)	12 (80)	3 (20)	
Above the median (n = 15, 50%)	11 (73)	4 (27)	
*Fi* ^*2*^	0.0062		
*p*	1		
Gender			
Male (n = 11, 63%)	8 (73)	3 (27)	
Female (n = 19, 37%)	15 (79)	4 (21)	
*Fi* ^*2*^	0.0050		
*p*	1		
Histopathological diagnosis			
Adenocarcinoma (n = 20, 67%)	17 (85)	3 (15)	
Squamous cell carcinoma (n = 10, 33%)	6 (60)	4 (40)	
*Fi* ^*2*^	0.78		
*p*	0.18		
Stage			
IIIB (n = 6, 20%)	6 (100)	0 (0)	
IV (n = 24, 80%)	17 (71)	7 (29)	
*Fi* ^*2*^	0.076		
*p*	0.29		
Smoking status			
NO (n = 4, 13%)	3 (75)	1 (25)	
YES (n = 26, 87%)	20 (77)	6 (23)	
*Fi* ^*2*^	0.00024		
*p*	1		
Immunotherapy line of treatment			
I (n = 7, 23%)	5 (71)	2 (29)	
II (n = 23, 67%)	18 (78)	5 (22)	
*Fi* ^*2*^	0.0047		
*p*	1		
PD-L1 (TC)			
<50% (n = 18, 60%)	13 (72)	5 (28)	
>/ = 50% (n = 12, 40%)	10 (83)	2 (17)	
*Fi* ^*2*^	0.017		
*p*	0.67		
Proton pump inhibitor usage			
NO (n = 22, 73%)	17 (77)	5 (23)	
YES (n = 8, 27%)	6 (75)	2 (25)	
*Fi* ^*2*^	0.00056		
*p*	1		
Toxicity			
No (n = 20, 67%)	16 (80)	4 (20)	
YES (n = 10, 33%)	7 (70)	3 (30)	
*Fi* ^*2*^	0.012		
*p*	0.66		
Response to immunotherapy			
PR + SD (n = 18, 60%)	13 (72)	5 (28)	
PD (n = 12, 40%)	10 (83)	2 (17)	
*Fi* ^*2*^	0.016		
*p*	0.67		
b) Continuous variables			
Characteristic	median	min-max	SD
PD-L1 (TC%), n = 30	30	0–95	29.4
BMI, n = 30	24.2	12.4–35.6	5.6
WBC [x 10^9/L], n = 27	7.4	3.6–102.0	18.4
CRP [mg/l], n = 26	32.5	0.9–128.5	32.2
LDH [U/l], n = 28	238.0	102.0–632.0	140.1
TSH [mlU/l], n = 19	0.76	0.13–4.24	1.09
Time between CTH and immunotherapy implementation (weeks), n = 23	16.3	1.9–53.6	14.8
Pack-years, n = 30	26.0	0–63	17.1

Abbreviations: BMI, body mass index; WBC, white blood cells, CRP - C-reactive protein, LDH, lactate dehydrogenase; TSH, thyroid-stimulating hormone; CHT, chemotherapy.

Disease stabilisation (SD) partial response (PR) and progression (PD) were reported in seven (23%), 11 (37%) and 12 (40%) patients, respectively. Response to immunotherapy, progression-free survival (PFS) and overall survival (OS), calculated from the immunotherapy administration, were assessed in all patients. The median PFS in the entire group was 15.4 months 95%CI: 5.5 to 23.0. PFS length data were available in 17 (57%) as complete and 13 (43%) as censored. PFS below and above 6 months was observed in 15 (50%) and 15 (50%) patients respectively. Median OS was 35.8 months (95% CI:13.0 to 35.8. OS length data were available in 15 (50%) as complete and in 15 (50%) as censored.

The study was performed with the consent of the Bioethics Committee of the Medical University of Lublin No. KE-0254/58/2019.

### Extraction and quality control of DNA

Stool samples were collected prior to immunotherapy implementation and stored at −80^o^C until DNA isolation. Samples were taken immediately after the therapeutic decision and before therapy administration. Two hundred mg of stool was used for extraction. The material was homogenized using a silica-based matrix (FastPrep, MP Biomedicals). Incubation was carried out at 37°C for 30 min with lytic enzymes: 20% lysozyme (10 mg/mL, A&A Biotechnology) and 10% lysostaphin (2 mg/mL, Sigma-Aldrich). DNA was extracted using Maxwell RSC (Promega) and the Maxwell^®^ RSC Tissue DNA Kit (Promega). Quality (parameters: A260/280 and A260/230) and quantity of total DNA were checked with a spectrophotometer (NanodropOne, Thermo Fisher Scientific) and fluorometer (dsDNA Broad Range, Qubit 3.0, Thermo Fisher Scientific), respectively.

### Libraries preparation, probe enrichment and sequencing

Libraries were prepared from 100 ng of total DNA using the Kapa HyperPlus Kit (Roche) with enzymatic fragmentation. The libraries were indexed using KAPA UDI Primer (Roche). Quality control of libraries was done by capillary electrophoresis (Fragment Analyzer, DNF-473 Standard Sensitivity NGS Fragment Analysis Kit 1–6,000 bp; Agilent) and fluorometric measurement (Qubit 3.0, Qubit Broad-Range Assay; Thermo Fisher Scientific). The libraries were enriched based on a panel of probes in accordance with the KAPA HyperCap Workflow v3.2 procedure (Roche). A panel of probes was custom-designed using the HyperDesign tool. Target was designed to be complementary to 6,094 known antimicrobial resistance genes (according to NCBI database - https://www.ncbi.nlm.nih.gov/bioproject/PRJNA313047, Accessed June 2022). The total target length was 5,723,224 bp. Short fragment sequencing was performed using a NextSeq sequencer (Illumina) in pair-end mode (V2.5, 2 × 150 bp) with PhiX as internal sequencing control.

### Data analysis

Raw data trimming and adapter removal were done by fastp (Chen, 2023). The detection of sequence targets from the targeted metagenome dataset was done by AMRPlusPlus ver. 2.0.0 (Bonin et al., 2023) with MEGARes database. Target detection was set at  ≥ 80% for coverage across the sequence length. The abundance of resistance mechanisms, classes, gene groups and genes were calculated based on the number of reads. Data is available at BioProject ID: PRJNA1068493.

### Statistics

Analyses were performed with Statistica 13 (TIBCO Software, California, United States) and MedCalc (MedCalc Software Ltd, Ostend, Belgium) software. The two-sided Fisher exact test was used to assess the differences between the treated patients and those not treated with antibiotics prior to immunotherapy. The U-Mann–Whitney test (U-M-W) was used to analyze differences in resistome composition (number of reads) according to dichotomous variables. Kaplan-Meier analysis was used to analyze PFS, and OS was assessed from the immunotherapy administration. A *p*-value below 0.05 was considered statistically significant, and the tendency to statistical significance was considered a range of 0.05–0.099.

## Results

The total number of reads per sample was between - 5852272 (230321_6836, PD group) and 34031176 (230629_7588, PR). The highest average number of resistome enrichment (average number of reads) and abundance of ARG populations were noted in the PD patients, while the lowest was in the PR patients (Supplementary Table S1). However, this data was not statistically significant.

The most commonly observed (over half of all reads) AMR determinants conferred resistance for tetracyclines (*tetQ*, *tetO*, *tetW*). Resistomes of patients across the study frequently showed the presence of resistance to MLS antibiotics (macrolides, lincosamides, streptogramins): *ermB* (erythromycin resistance in particular), *mefA*, *mefE*, *msrD*, *lnuA* (particularly lincosamide resistance), beta-lactamases (*bla*_TEM_), including cephalosporinases (*cfx, CblA, bla*_EC_ *, cepA, bla*_SHV_ *, bla*_OXA_, *AmpC, bla*_CMY_), glycopeptides (*vanD, vanHD, vanRD, vanSD, vanYD*) and aminoglycosides (via *ant(6), aph(3), aph(2), aac6, ant(9*).

**FIGURE 2:**
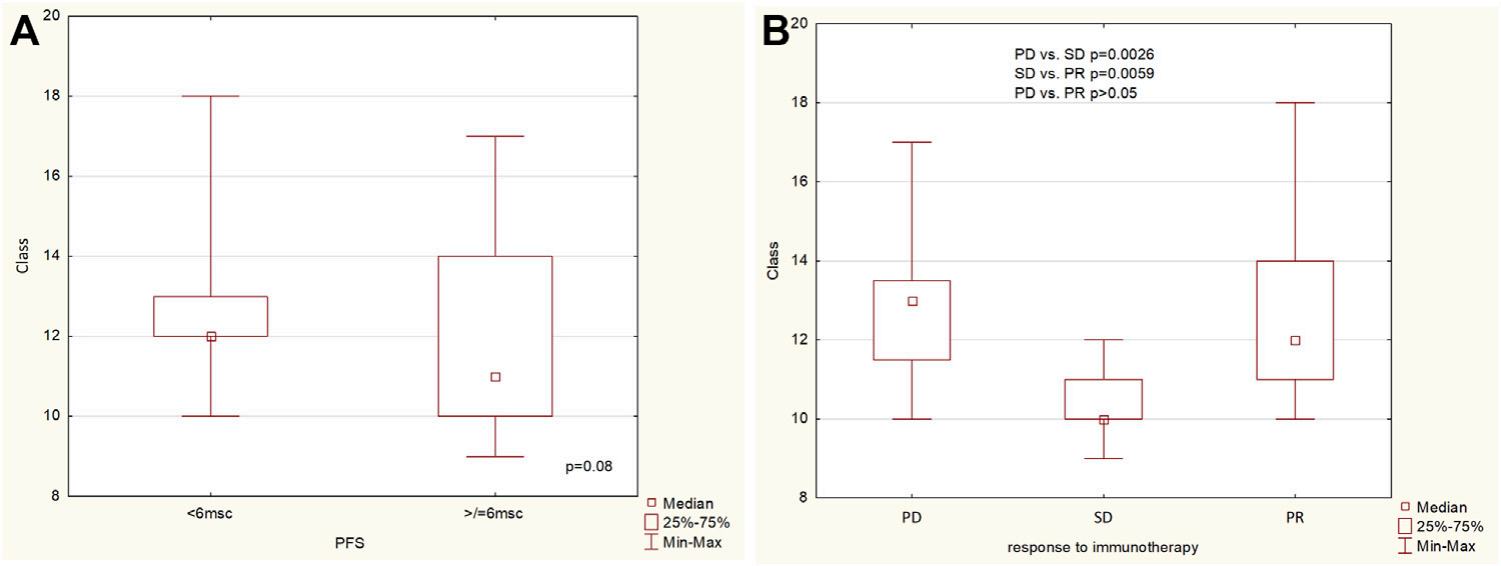
The richness of ARG classes **(A)** in the group of patients with PFS below and above 6 months, **(B)** in the group of patients with progression disease (PD), stable disease (SD) and partial response (PR).

The richness (the number of observed categories) of patients’ resistome was assessed on multiple levels: gene, gene groups, mechanisms and classes of antimicrobial resistance. Resistome richness at the class level was higher in the group of patients with PFS less than 6 months, with a trend towards statistical significance (*p* = 0.08, Figure 2A). Statistically differences were found at the class level between PD vs. SD and PR vs. PD groups, and the difference in richness between PR and PD was not statistically significant (*p* > 0.05) (Figure 2B).

Spearman’s analysis showed that the frequency of observed gene groups in multiple cases correlates with the clinical parameters of NSCLC patients treated with ICI. Table 2 shows the level of correlation (R) and *p*-value of a particular group of genes according to the analyzed clinical features. We observed a negative correlation between PFS and an abundance of gene groups conferring resistance to vancomycin - *vanHD* (vanH variant in the vanD gene cluster) and lincosamide - *lnuC* (nucleotidyltransferase - *lnu*). Further, OS was negatively correlated with resistance to vancomycin (*vanSD* and *vanYD*), tetracyclines - *tetQ*, macrolide (*mefA*, *msrD*), MLS groups (*ermG*) and aminoglycosides (*aph(*6) - phosphotransferase enzymes with modification at the 6-hydroxyl group of the antibiotic). Similarly, the association was shown in the case of TSH (thyroid-stimulating hormone) with class A beta-lactamases that confer resistance to cephalosporins - *CblA*. LDH (lactate dehydrogenase) was negatively correlated with tetracycline resistance (*tet40* gene group) and positively correlated with cephalosporinases marker - *cepA*. Time from CHT (chemotherapy) to immunotherapy was positively correlated with *cfr* (23S ribosomal RNA methyltransferase), conferring resistance to drug classes: lincosamide, oxazolidinone, phenicol and streptogramin antibiotics via target alteration. The CPR factor was positively correlated with detected levels of O-nucleotidyltransferases–*ant(9*), providing resistance to aminoglycoside antibiotics; plasmid-mediated nucleotidyltransferase - *lnuA* and *mefE* - a proton motive efflux pump that confers resistance to macrolides. The pack-years (total number of packs of cigarettes smoked during a year) factor was positively correlated with multiple gene groups - *vanD*, *vanHD*, *vanSD* and *vanYD* associated with vancomycin resistance.

**TABLE 2:** Results of Spearman’s correlation analysis between clinical features expressed on a continuous scale and ARGs groups.

	Clinical features						
	Pack-years, n = 26	LDH, n = 28	CRP, n = 26	TSH, n = 19	Time (weeks) from CHT to immunotherapy, n = 23	PFS (months), n = 30	OS (months), n = 30
Group of genes	*vanD*, R = +0.55, *p* = 0.0018 *vanHD*, R = +0.54, *p* = 0.0018 *vanSD*, R = +0.45, *p* = 0.012 *vanYD*, R = +0.45, *p* = 0.011	*tet40*, R = −0.42, *p* = 0.023 *cepA*, R = +0.39, *p* = 0.042	*ant*(9), R = +0.44, *p* = 0.023 *lnuA*,R = +0.44, *p* = 0.026 *mefE*, R = +0.42, *p* = 0.032	*CblA*, R = −0.48, *p* = 0.034	*cfr*, R = +0.51, *p* = 0.013	*vanHD*, R = −0.44, *p* = 0.015 *lnuC*, R = −0.42, *p* = 0.020	*tetQ*, R = −0.42, *p* = 0.020 *vanSD*, R = −0.37, *p* = 0.043 *vanYD*, R = −0.37, *p* = 0.044 *aph*(6), R = −0.54, *p* = 0.0019 *ermG*, R = −0.44, *p* = 0.016 *mefA*, R = −0.44, *p* = 0.015 *msrD*, R = −0.580, *p* = 0.00073

Abbreviations: Pack-years - multiplying the number of packs of cigarettes smoked per day by the number of years, LDH, lactate dehydrogenase, CRP - C-reactive protein, TSH, thyroid-stimulating hormone; CHT, chemotherapy; PFS, progression-free survival; OS, overall survival.

The abundances of several ARGs were assisted with clinicopathological features: antibiotic use before immunotherapy, histopathological diagnosis, smoking status, toxicity related to immunotherapy, and response to immunotherapy or PFS (Mann-Whitney U test analysis shown in Table 3).

**TABLE 3:** Results of Mann-Whitney U test analysis between clinical features expressed as a dichotomous variable and abundance of resistance gene group.

Clinical features	Gene name, *p-value*	Description
Antibiotics use before immunotherapy	*CblA, p = 0.048 ermR, p = 0.042* *vanXD, p = 0.042*	Higher abundance in patients treated with antibiotics
	*AmpC*, *p* = 0.011 *bla*_EC_, *p* = 0.033	Higher abundance in patients not treated with antibiotics
Histopathological diagnosis	*tetX*, *p* = 0.044 *vanD*, *p* = 0.0032 *vanHD*, *p* = 0.0016 *vanSD*, *p* = 0.00062 *vanYD*, *p* = 0.00050	Higher abundance in SqC
Smoking status	*ermG*, p = *p* = 0.0028 *msrD*, *p* = 0.014 *vanD*, *p* = 0.014 *vanHD*, *p* = 0.0069 *vanSD*, *p* = 0.0088 *vanYD*, *p* = 0.011	Higher abundance in smokers
Toxicity related to immunotherapy	*aph*(3), *p* = 0.035 *bla*_EC_, *p* = 0.0072	Higher abundance in patients without toxicity confirmed
Response to immunotherapy	*lnuC*	Higher abundance in patients with PD in comparison to PR + SD (*p* = 0.035) and to PR (*p* = 0.0056)
	*bla*_EC_, *p* = 0.045 *fosA*, *p* = 0.022	Higher abundance in patients with PD in comparison to SD
	*vanRD*, *p* = 0.0012	Higher abundance in patients with SD in comparison to PR
PFS	*tet32*, *p* = 0.045	Higher abundance with PFS below 6 months

In patients treated with antibiotics less than 4 weeks before immunotherapy, significantly higher levels of gene groups were observed: *ermR* - element of the erythromycin biosynthetic cluster and *vanXD* - glycopeptide resistance gene cluster. Further, while high levels of A class beta-lactamase - *CblA* were noted in patients regardless of antibiotic therapy status (median = 51471 reads), significantly higher level of this cephalosporinase gene group was seen in patients treated with antibiotics (median = 11926 reads). Additionally, statistically significant levels of C-class beta-lactamases *AmpC* and *bla*_EC_ (conferring resistance to cephalosporins) were observed in patients not treated with antibiotics up to 4 weeks before ICI. Those gene groups were less abundant in patients treated with antibiotics.

Histopathological diagnosis indicates a higher abundance of resistance markers to tetracycline (*tetX*) and vancomycin (*vanD*, *vanHD*, *vanSD* and *vanYD*) in SqC.

Patients who were smokers had a significantly higher abundance of gene groups coding resistance to vancomycin: *vanD*, *vanHD*, *vanSD*, *vanYD* and macrolides: *ermG* and *msrD*.

A high abundance of C-class beta-lactamases, *bla*_EC_ and *AmpCR*, and aminoglycoside O-phosphotransferases - *aph*(3) was observed in patients without confirmed toxicity.

The higher abundance of *lnuC* were observed in PD patients than in PR and PR + SD. Further, PD patients, compared to SD, had a significantly higher abundance of genes conferring resistance to cephalosporins - *bla*_EC_ and fosfomycin - *fosA*. On the other hand, SD patients showed increased levels of *vanRD* compared to PR. Additionally, a higher abundance of *tet32* in patients with progression-free survival below 6 months was noted.

**FIGURE 3:**
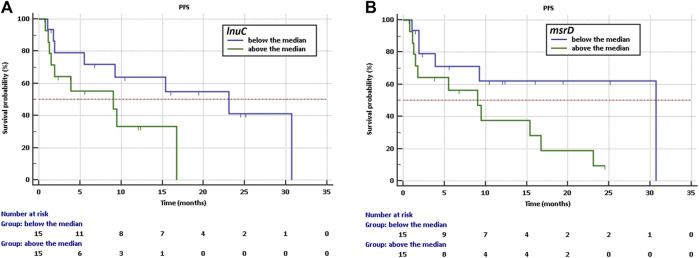
Kaplan-Meier survival curves of PFS for NSCLC patients treated with immunotherapy depending on **(A)** abundance of *lnuC*, **(B)** abundance of *msrD*.

The Kaplan-Meier analysis showed that a high abundance of ABC efflux pump - *msrD* and lincosamide nucleotidyltransferases - *lnuC* indicate a higher risk of shorter PFS with HR = 2,59, CI: 0,96 to 6,94 and HR = 2,64, CI: 0,91 to 7,70 respectively. Longer PFS was observed if the abundance was below the median in the case of *msrD* (30,7 vs. 9 months, *p* = 0.059, Figure 3A) and the case of *lnuC* (23.0 vs. 9 months, *p* = 0.074, Figure 3B).

Further, high abundance of *ermG* (rRNA adenine N-6-methyltransferase), *aph*(6) (aminoglycoside O-phosphotransferase), *fosA* (fosfomycin thiol transferases) and *msrD* indicate shorter OS with HR = 2.75, 95%CI: 0.98–7.72, HR = 2.79 CI: 0.99–7.8, HR = 2.60, 95%: 0.93–7.25 and HR = 4.10, 95%CI: 1.43–11.62, respectively.

**FIGURE 4:**
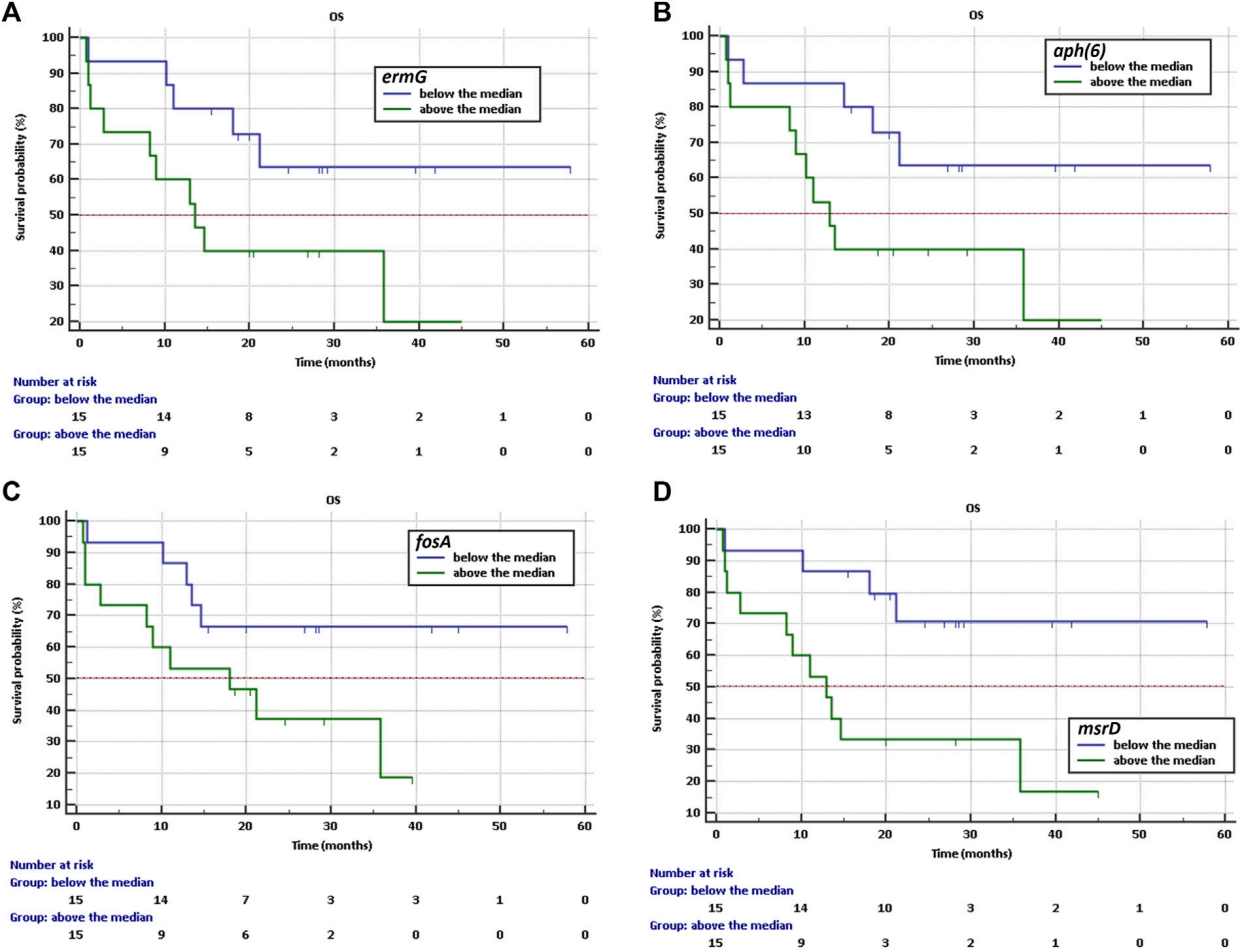
Kaplan-Meier curves of overall survival curves of OS for NSCLC patients treated with immunotherapy depending on **(A)** abundance of *ermG*, **(B)** abundance of *aph*(6), **(C)** abundance of *fosA*, **(D)** abundance of *msrD*.

Longer OS was observed if the abundance was below the median for *ermG* (not reached vs. 13.5 months, *p* = 0.055, Figure 4A), for *aph*(6) (not reached vs. 13.0 months, *p* = 0.052, Figure 4B), for *fosA* (not reached vs. 18.0 months, *p* = 0.068, Figure 4C) and for *msrD* (not reached vs. 18.0 months, *p* = 0.0084, Figure 4D), respectively.

### Discussion

The percentage of tumor cells with PD-L1 expression is currently the only factor for qualifying patients for immunotherapy (Gandhi et al., 2018). Unfortunately, this is not a perfect marker, and patients’ responses to immunotherapy cannot always be accurately predicted (Topalian et al., 2019; West et al., 2019). In some patients with a high percentage of tumor cells expressing PD-L1, treatment with anti-PD-1 or anti-PD-L1 antibodies may be ineffective and primary or secondary resilience could occur. Immunotherapy may benefit patients with a low percentage of TC or no expression of this protein on cancer cells (Gandhi et al., 2018; Vokes et al., 2018; Pacheco et al., 2019; Topalian et al., 2019; West et al., 2019; Mustieles and Fernández, 2020). Therefore, additional factors are being sought to predict response to immunotherapy in patients with NSCLC.

Genetic factors, such as the occurrence of mutations in the *KRAS (Kirsten Rat Sarcoma Viral Oncogene Homolog), BRAF (B-Raf Proto-Oncogene, Serine/Threonine Kinase), STK11 (Serine/Threonine Kinase 11), KEAP1 (Kelch Like ECH Associated Protein 1)* genes, and epigenetic factors such as methylation levels or microRNA expression also draws the attention (Boyero et al., 2020). This trend also includes the composition of the intestinal microbiome and the occurrence of fastidious bacterial species (Grenda et al., 2022; Sun et al., 2023; Li et al., 2024).

Studies point at particular groups (e.g., Ruminococcaceae*, Clostridia Enterococaceae,* Bifidobacteriaceae) and species of bacteria (e.g., *Akkermansia muciniphila, Enterococcus hirae, Faecalibacterium prausnitzii, Barnesiella intestinihominis*) as being potentially associated with patients response to immunotherapy treatment (Daillère et al., 2016; Gharaibeh and Jobin, 2019; Griffin et al., 2021; Oh et al., 2021; Derosa et al., 2022; Procaccianti et al., 2023). Most metagenomic studies focus on microbiome taxonomic composition and do not take under consideration the genomic load (including resistance genes) of bacteria or do it minimally (Lanza et al., 2018; Stege et al., 2022; Fredriksen et al., 2023; Shay et al., 2023; Xi et al., 2023). Our study focuses on the resistome - a collection of antibiotic resistance genes (ARGs) in a gut microbiome. We suggest that his new culture-free approach enables targeted and sensitive assessment of ARG load in the microbiomes of patients and its potential association with response to immunotherapy.

One of leading cause of death in patients with cancer is hospital infections, particularly with the ESKAPE pathogens: *Enterococcus faecium, Staphylococcus aureus, Klebsiella pneumoniae, Acinetobacter baumannii, Pseudomonas aeruginosa,* and *Enterobacter spp*. (Nanayakkara et al., 2021). Ineffective antibiotic treatment due to multidrug resistance (MDR) in hospital settings is mostly associated with methicillin-resistant *S. aureus* (MRSA), vancomycin-resistant *enterococci* (VRE), Extended-spectrum beta-lactamase (ESBL)-producing Enterobacteriaceae (examples of common Enterobacteriaceae are *Escherichia coli* and *K. pneumoniae*), multidrug-resistant *P. aeruginosa*, or *Clostridium difficile* (van Duin and Paterson, 2020). Resistant bacteria can negatively affect NSCLC patients by inducing chronic infection (with the majority of them being pneumonia), usually caused by Gram-negative Enterobacteriaceae or MRSA (Bavaro et al., 2021). Moreover, prolonged infections, resulting in antibiotic treatment supplemented by probiotics, precisely those containing resistant bacteria strains (due to the potential for horizontal gene transfer), may impact the microbiome and resistome of immunocompromised patients, possibly causing further alterations (Bonnet et al., 2023; Sun et al., 2023; Li et al., 2024). We found a correlation between multiple acquired genes, conferring resistance to aminoglycosides, beta-lactams (cephalosporin in particular); macrolides, glycopeptides (particularly vancomycin) and tetracyclines antibiotic classes to several clinical features of characterized NSCLC patients treated with immunotherapy.

Predictably, an important clinical feature that impacted resistome composition in our study was antibiotic (AB) use. These observations followed multiple studies, including meta-analysis, showing the negative impact of exposure to ABs for cancer patients during the treatment with immune checkpoint inhibitors (ICIs). ATB-induced dysbiosis might influence the clinical response by modulating the gut microbiome (Elkrief et al., 2019). Antibiotic treatment applies intense selection pressure for acquiring and maintaining ARGs on the bacterial community, driving the observable expansion of the disease-associated resistome (Fredriksen et al., 2023). AB use may impair the effectiveness of ICI treatments, resulting in inadequate patient outcomes, with AB use significantly reducing OS and PFS (Petrelli et al., 2020; Crespin et al., 2023). Specifically, exposure shortly before or after ICI initiation seems to have a significant impact (Lurienne et al., 2020). The resistome appears to affect multiple diseases. However, to our knowledge, this is the first study showing a direct link between the particular genomic composition of the resistome and the outcome of NSCLC immunotherapy (Fredriksen et al., 2023). This thesis is further supported by data that links antibiotic treatment and several resistance gene groups.

We noted that in patients treated with antibiotics directly before ICIs, the resistome seems to have significant numbers of genes conferring resistance to vancomycin (via *vanXD*, VanD cluster) and macrolides (via *ermR*). Vancomycin-resistant bacteria (VRB), mainly *Enterococci* and *Staphylococci* can cause life-threatening infections that are increasingly complex to treat (Selim, 2022), especially when vancomycin is a first-line antibiotic used to treat invasive MRSA infections. Fortunately, community-acquired vancomycin resistance in pathogenic bacteria that may threaten public health, such as *S. aureus* and *C. difficile* are still rare (Palmieri et al., 2023). *Enterococcal* isolates carrying *vanD* gene clusters have been commonly reported worldwide and phenotypically seem moderately resistant to vancomycin and teicoplanin (Fang et al., 2007; Brochu et al., 2023). VanXD gene group, in particular, is associated with *E. faecium, Enterococcus faecalis, Blautia product end Enterocloster clostridioformis* (Alcock et al., 2023). Acquiring the v*anRD, vanSD, vanYD, vanHD, vanD,* and *vanXD* clusters of genes results in VanD-type vancomycin resistance in Enterococci (Brochu et al., 2023). We noted that genes of the VanD cluster seem to be linked with antibiotic use and multiple other factors: smoking, histopathological diagnosis, response to immunotherapy (*vanRD* - higher abundance in SD patients) and PFS.

We also observed a link between antibiotic therapy and resistance to macrolides via *ermR* - detected in multiple gram-positive bacteria, but mainly linked with erythromycin-resistant *Streptococcus agalactiae* strains (Domelier et al., 2008; Alcock et al., 2023). Macrolides are broad-spectrum antibiotics regarded as critically important in human and veterinary medicine. They share sites of action (50S ribosomal subunits) and resistance mechanisms with lincosamides and streptogramins, thus named the MLS group (Schroeder and Stephens, 2016). Resistance to MLS antibiotics is mostly due to acquiring erythromycin resistance methylase (*erm*) genes (Schmitz and Fluit, 2010). Macrolide resistance may occur due to three mechanisms: ribosomal demethylation - catalysed by erythromycin ribosomal methylase (*erm*), two-component efflux pump - coded by genes *msr*D/*mefE* or ribosomal mutations (Schroeder and Stephens, 2016).

In addition, antibiotic therapy was associated with significant numbers of bacteroides-specific cephalosporinase - *CblA*, naturally produced by various genera: *Bacteroides*, *Capnocytophaga*, *Chryseobacterium*, and *Elizabethkingia* (Philippon et al., 2016; Alcock et al., 2023). Interestingly, resistomes of patients not treated with antibiotics up to 4 weeks before ICI’s treatment had very high abundance of cephalosporinases - C classes (*AmpC* and *bla*_EC_) encoded in the genome of multiple Gram-negative bacteria, including *E. coli, Citrobacter* spp.*, Serratia* spp.*, E.* spp.*, and P. aeruginosa* (Alcock et al., 2023). It may be due to several reasons: longer time of persistence of those cephalosporinase genes after previous antibiotic treatment, cross-contamination from the environment - e.g., home or hospital, especially with former imputing selective solid pressure to generate resistant strains, as well due to food contamination with resistant strains (EFSA and ECDC, 2022; WHO and ECDC, 2022; Shay et al., 2023).

Several clinical and epidemiological studies indicated that cigarette smoke exposure can significantly increase the usage of antibiotics due to increased pulmonary infections, confirming an increased risk of multi-drug-resistance proliferation within the human lung and natural environments (Xu et al., 2020; Fang et al., 2023). It directly enhanced the virulence of a common human pathogen, *S. aureus*. Cigarette smoke promotes the binding of multiple pathogens, such as *Neisseria meningitidis, Streptococcus pneumoniae*, and *S. aureus*, to epithelial buccal cells (Chien et al., 2020). Our study confirms this, showing an association between smoking and ARG’s conferring macrolides - *ermG* and *msrD*, as well as genes coding resistance to vancomycin (vanD gene cluster). It was further confirmed also by the positive correlation between various genes from the vanD cluster and the pack-years factor. Evidence in the literature indicates that smoking may impede the efficacy of macrolide-based antimicrobial therapy by accelerating the onset and magnitude of *ermB*-mediated resistance (Matapa et al., 2019). A recent *in vitro* study indicated that cigarette smoke increases the twofold transfer of MDR-plasmids between *Pseudomonas* strains in an artificial lung sputum medium, suggesting a similar mechanism *in vivo* (Fang et al., 2023). Typical usage of macrolides has been associated with increased macrolide resistance in *S. pneumoniae* (primarily due to the *ermB* gene) and clinical treatment failures.

The resistome of NSCLC patients seems to show significant differences depending on the response to immunotherapy, with the resistance of PD patients being richer and more abundant. A study by Xi et al. using direct metagenomics to evaluate the microbiome of NSCLC patients treated with chemotherapy also indicated that the short-PFS patients had a trend with more antibiotic resistance genes than healthy control and long-PFS groups (Xi et al., 2022). We also observed that the resistome of PD patients harbored significantly higher numbers of the *lnuC* gene group, which confers unique resistance to lincosamides (L phenotype). The production of nucleotidyltransferases (*lnu*) is uncommon among Gram-positive bacteria and has been detected mainly in clinical isolates of *S. agalactiae*, *S. anginosus,* and more recently *Lactobacillus species* isolates from human gut (Achard et al., 2005; Gravey et al., 2013; Moradi et al., 2022). Lincosamides are a class of antibiotics that can be helpful against infections that are aggressive or resistant to other treatments, e.g., caused by *Staphylococcus bacteria*, including Methicillin-resistant *S. aureus* (MRSA) (Bryskier, 2010).

On the other hand, the resistome of SD patients was characterized by a significant number of cephalosporinases, *bla*_EC_ and fosfomycin resistance coded via *fosA*. Variants of *bla*_*EC*_ genes are the most abundant C-class beta-lactamases (representing almost half of all described beta-lactamases), with significant clinical relevance that shows an extended capacity to act on higher-generation cephalosporins (Naas et al., 2017; Schmidt et al., 2023). Genes *bla*_EC_ are widely distributed in such bacteria as *Klebsiella* spp.*, E.* spp., *Acinetobacter* spp.*, or Pseudomonas* spp., thus creating a significant challenge in the treatment of infections caused by MDR-pathogen (Ito et al., 2017; Schmidt et al., 2023). Following increased resistance to quinolone and beta-lactam antibiotics, fosfomycin usage has increased in clinical settings (Oteo et al., 2010). Fosfomycin is a broad-spectrum antimicrobial drug that is the most used first-line therapeutic agent for urinary tract infections due to its high activity on a broad spectrum of bacteria (Güneri et al., 2022). Resistance rates to fosfomycin have been reported at low to moderate levels among pathogenic Enterobacterales, but it has recently been increasing (Güneri et al., 2022). Cases of *fosA* gene detection in *E. coli*, while still rare, appear to be more common, especially in the context of hospital environments, often in co-resistance with epidemiology-relevant cephalosporinases variants - *bla*_CMY-2_ *, bla*_CTX-M-14_ *, bla*_CTX-M-15_ *, bla*_SHV-12_ (Oteo et al., 2010; Güneri et al., 2022).

We noted that genes connected to the resistance to tetracyclines were very abundant in the resistome of NSCLC patients. It seems to be in accordance with the results of (Xi et al., 2022), which characterized the gut microbiota of NSCLC patients treated with concurrent chemoradiotherapy. They noted *tetQ* (mainly originating from *Bacteroides*) as most abundant in the resistome of shot-PFS patients compared to long-PSF. In the context of PFS, we did not observe statistically significant differences in *tetQ* abundance. However, we noted a negative correlation between this gene group and OS. Further, we noted that short-PFS (below 6 months) was associated with the *tet23*. Tetracyclines are well-known and commonly used antibiotics with a broad spectrum of activity, interacting with Gram-positive and -negative bacteria, spirochetes, obligate intracellular bacteria, and protozoan parasites (Grossman, 2016). Both underlined above *tet* gene variants are ribosomal protection proteins that bind to the ribosome to induce tetracycline resistance (Alcock et al., 2023). They were linked with multiple mobile genetic elements, e.g., IS21 family transposase and various gram-positive and -negative plasmids, thus their significant potential for horizontal gene transfer (HGT) across bacteria community (Tribble et al., 2010; Bartha et al., 2011). *Prevotella* and *Bacteroides* are considered as main reservoirs of those genes, with *tetQ* commonly detected (from WGS data) in *Bacteroides caccae, B. fragilis, B.s ovatus, B.s thetaiotaomicron, Parabacteroides distasonis, Phocaeicola dorei, P. vulgatus, Prevotella bivia* and *P. intermedia,* and *tet32* in Lachnospiraceae *bacterium, Ruthenibacterium lactatiformans, Streptococcus anginosus, Streptococcus pseudopneumoniae, S. suis* and *Clostridium*-related human colonic anaerobes (Melville et al., 2001; Alcock et al., 2023). Further, both genes were shown to be associated with oral and human gut microbiome (Melville et al., 2001; Warburton et al., 2009; Tribble et al., 2010). Hsieh et al. indicate that co-administration of EGFR-TKIs and tetracyclines (TC) improved the PFS and OS. Skin rashes, which are a side effect of using EGFR-TKIs, can be relieved by TC. The authors suggest that the prolongation of PFS or OS is not due to the direct impact of TC but to the effect of antibiotics relief of the skin rash and the possibility of continuing TKI therapy (Hsieh et al., 2023).

Our study indicates a negative correlation between progression-free survival time and levels of previously described *lnuC* and *vanHD*, which are associated with resistance to lincosamide and vancomycin, respectively. Further, an increase in markers of lincosamide - *lnuC* and macrolide - *msrD* resistance indicates a risk of shorter PFS. The increase in the numbers of macrolide (*ermG* and *msrD*) and fosfomycin (*fosA*) resistance were associated with shorter OS. It underlines a strong link between resistance to vancomycin and MLS group antibiotics and PFS and OS of NSCLC patients. Macrolides are used to treat multiple respiratory conditions: pneumonia, sinusitis, pharyngitis, and tonsillitis, bronchiectasis, particularly for patients demonstrating recurrent and persistent exacerbations (Patel and Hashmi, 2023). Several studies showed macrolide resistance, including efflux *msrD*(*mel*), rRNA methylases *ermB*, *ermX*, and *ermF* genes, as a base of sewage resistome, human gut and respiratory microbiome (Roberts, 2011; Ohashi and Fujisawa, 2017; Hendriksen et al., 2019; Sukumar et al., 2023). Increased abundance of resistance genes from the Macrolide–Lincosamide–Streptogramin (MLS) group has been dominant in airways associated with multiple respiratory diseases. This “core resistome” is often characterized by the predominance of pathogenic bacteria such as *Haemophilus* spp., *Streptococcus* spp., and or *P.* spp., forming “airway pathobiome” (Sukumar et al., 2023). Macrolide resistance is among the most prevalent in the broader environment, representing a rich source of resistance determinants with a strong potential for horizontal transfer (Hendriksen et al., 2019). We also noted that sequences coding resistance to vancomycin (vanD gene cluster) were negatively correlated with PFS. It seems to be opposite to the results of Xi et al., who observed significantly more of this gene group in the long-PFS group compared to the short-PFS group (Xi et al., 2022). However, the studies need to consider several differences: biological differences between the patients, probably different AB treatments, size of the study (Xi et al. n = 18 NSCL patients), and differences in applied therapies. Studies in an enlarged group of patients are needed, divided into patients treated in the first line with monotherapy or chemoimmunotherapy and treated with ICIs in the second line compared to chemotherapy alone. Referring to Hsieh’s research, examining the resistome profile in patients treated with molecularly targeted therapies would also be necessary.

### Conclusions

The study showed a direct link between antibiotic treatment and the shape of the resistome of NSCLC patients via markers of vancomycin (vanD cluster) and cephalosporin resistance (*CblA*). Further, cephalosporinases (*AmpC* and *bla*_EC_) persist even in the absence of AB treatment up to a month before ICIs. We noted the impact of smoking on the increased abundance of MLS and vancomycin core resistome. Resistome of PD patients seems to be more abundant and diverse, with significantly higher levels of genomic markers of resistance to lincosamides (*lnuC*), cephalosporins (*bla*_EC_) and fosfomycin (*fosA*). Progression-free and overall survival time were linked with increased levels of MLS (*mefA*, *ermG*, *msrD*, *lnuC*), tetracycline (*tetQ/32*) and vancomycin (*vanS/YD*) resistance gene groups. Our work highlights the versatility and sensitivity of targeted metagenomics in assessing functional aspects of the human microbiome, including the phenomenon of antibiotic resistance. The data demonstrated the existence of an association between antibiotic use, smoking, response to ICIs and PFS/OS of NSCLC patients and multiple acquired ARGs - mainly responsible for resistance to cephalosporins, MLS and vancomycin. It is probably related to patients’ overall health, disease progression, prolonged infections and AB treatments.

Further, some of the resistance markers noted in the study have been associated with bacterial species and resistome connected with multiple airway diseases and chronic infections. Some observed dependences can potentially be a prognostic factor in NSCLC treatment and accompanying targeted antibiotic treatments as needed, e.g., in cases of MDR infections. However, what needs to be determined is if modification of resistome and microbiome (e.g., via diet or/and non-AMR/AMR probiotics) may positively impact ICI treatment and patient prognosis.

## Data Availability

The datasets presented in this study can be found in online repositories. The names of the repository/repositories and accession number(s) can be found in the article/Supplementary Material.
